# Inhibitory Control of Emotional Interference in Deaf Children: Evidence From Event-Related Potentials and Event-Related Spectral Perturbation Analysis

**DOI:** 10.3389/fpsyt.2022.897595

**Published:** 2022-06-24

**Authors:** Qiong Chen, Junfeng Zhao, Huang Gu, Xiaoming Li

**Affiliations:** ^1^Shaanxi Provincial Key Research Center for Children Mental and Behavioral Health, School of Psychology, Shaanxi Normal University, Xi'an, China; ^2^Institute of Behavior and Psychology, School of Psychology, Henan University, Kaifeng, China; ^3^Department of Health Promotion, Education, and Behavior, University of South Carolina, Columbia, SC, United States

**Keywords:** deaf children, interference control, emotional stroop, event-related potentials, time-frequency analysis

## Abstract

**Background:**

Impairment of interference control ability may reflect a more general deficit in executive functioning, and lead to an increase in internal-externalized problems such as impulsivity, which has been reported in deaf children. However, few researches have examined the neural mechanism of this impairment.

**Methods:**

This study applied the electroencephalogram (EEG) technique to investigate the interference control ability in 31 deaf children and 28 hearing controls with emotional face-word stroop task.

**Results:**

Results from behavioral task showed that deaf children exhibited lower accuracy compared to hearing controls. As for EEG analysis, reduced activation of ERP components in N1 and enhanced activation of ERP components in N450 have been found in deaf children. Besides, incongruent condition elicited larger N450 than congruent condition. Furthermore, for brain oscillation, alpha band (600–800 ms) revealed a reduced desynchronization in deaf children, while theta band (200–400 ms) revealed an enhanced synchronization in deaf children and incongruent condition, which were in line with ERP components.

**Conclusion:**

The present findings seem to indicate that the deficit during emotional interference control ability among deaf children might be due to the impaired attention allocation ability and emotional cognitive monitoring function during emotional conflict detection process. Consequently, reduced N1 and enhanced N450 might be due to early attention impairment causing more effort of deaf children later in emotional cognitive monitoring.

## Introduction

The World Health Organization (WHO) estimates that there are approximately 360 million people with hearing impairment in the world and almost one-tenth of the affected population are children (1). Previous research has shown that hearing loss not only affects the normal development of language skills, but also affects other neurocognitive functions among deaf children, such as interference control ability (2, 3). However, most of these previous studies were done with questionnaires or behavioral experiments (4–6) and focused on the performance of working memory, attention, inhibitory control and other executive functions of deaf children. Few of them ever implied electroencephalogram (EEG) technique to investigate the neural mechanisms of this interference control impairment among deaf children. However, EEG signals, with their millisecond temporal resolution, are excellent at tracking rapid changes in brain function, and techniques to acquire these signals are relatively simple and non-invasive, providing more accurate and detailed information to help estimate inhibitory control. Therefore, this study selected it to identify the inhibitory control of emotional interference in deaf children (7, 8).

In addition to the commonly studied interference control ability, various emotion skills are also believed to be impaired in deaf children. For instance, facial emotion processing, as one of the most studied aspects of social cognitive function, is reported being impaired in deaf children (4, 9, 10). Moreover, dozens of studies also showed that deaf children are more challenged in terms of emotion identification, emotion understanding, and the expression of emotion compared with hearing controls (11–16). Such challenges can be attributed to delayed language acquisition or lack of personal experience opportunities to talk with others, as well as the long-term stress environment in which they are trapped in emotional states such as anxiety, depression and subjective anxiety.

Moreover, Gray (17, 18) believed that cognition and emotion are strongly integrated and inseparable in the process of information processing (19). A meta-analysis of inhibitory control demonstrated that several brain areas have been associated with the mechanisms underlying inhibitory control, with a network involving left and right inferior frontal gyrus (IFG) dorsolateral pre-frontal cortex (dlPFC), anterior cingulate (ACC) (20). Specifically, the role of the anterior cingulate cortex (ACC) and the dorsolateral pre-frontal cortex (DLPFC) regions have been shown to be components of a neural network which plays a critical role in the completion of tasks requiring self-monitoring and inhibition (21, 22). In addition, several meta-analyses of emotion regulation reported activations in the bilateral dorsolateral pre-frontal cortex (dlPFC), ventrolateral pre-frontal cortex (vlPFC), dorsal anterior cingulate cortex (dACC) (23–25), which largely overlaps with the classic frontoparietal cognitive control network (26).

According to the above evidence analysis (2–4, 9, 10, 20, 23–26), we can know that emotional and inhibitory control disorders both exist in deaf children, and the activation brain regions of cognitive and emotional networks are highly overlapped. Therefore, the present study combined two aspects and employed the face-word emotional stroop task to investigate the emotional inhibitory control of deaf children (27–29). In this task, “happy” and “fear” words with red color are superimposed across facial expressions of happy and fear, conflict effects occur when emotional words and facial expressions are incongruent, which has been widely used to examine the inhibitory control of emotional interference (28, 29). Previous studies have observed two important ERP components that were related to emotional interference control processing: N1 and N450 (28, 30–39). The N1 component of the ERP reflect brain activation in the early perceptual stages (38). It was hypothesized that larger amplitudes of the sensory components (N1) to emotional words indicated an increased attention-related cerebral processing during relatively early perceptual stages of information processing (31). The N450 is a popular index of conflict detection in emotional conflict control tasks which shows larger negative amplitude in the incongruent condition compared to congruent condition (34–37).

Furthermore, time-frequency analysis (TFA) can provide complementary information on neural processing dynamics that is distinctive from traditional phase-locked ERP method. Therefore, according to previous studies, theta and alpha band are used for analysis to explore the characteristics of emotional suppression control in deaf children (40–46). The frontal-central distribution of the evoked theta (4–7 Hz) response is suggested to be related with central executive and working memory processes, and reflects initiation of the central executive processes to detect interference and to inhibit the response for task-irrelevant features (40, 42, 43, 47). Alpha desynchronization (8–14 Hz) which reflects attentional processes, processing of sensory–semantic information and the difficulty of the task, that is to say, the more demanding a task, the stronger the amount of event-related alpha desynchronization (40, 44–46).

Taken together, inhibitory control of emotional interference is vital not only for good behavior and cognitive function, but also for adequate emotional control and social interaction (48, 49). In the current study, we applied EEG technique to investigate both emotion and cognitive abilities using face-word emotional stroop task, and further explore the potential neural markers of emotional interference control deficit among deaf children. On the basis of previous research (10, 48, 50–52), our hypothesis was that compared to hearing controls, deaf children would show worse performance in both behavioral and EEG measures during emotional stroop task.

## Materials and Methods

### Participants

We performed a sample size calculation on the basis of G^*^Power 3.1, using an alpha level of 0.05 with 95% power to detect a large effect size (f = 0.4). Results showed that a sample size of 12 would be needed to assure the adequate statistical power. Therefore, a total of 31 deaf children aged 9–13 were recruited from the Kaifeng Special Education School in central China and 28 matched hearing controls were recruited from the same geography area. Parent and teacher reports established that all children were born to hearing parents and had no apparent mental health disorders such as ADHD or autism spectrum disorders. Main demographic characteristics of both deaf children and hearing controls were shown in Table 1. Participants were required to complete a computer version of emotional conflict task while recording EEG. Each participant received an age-appropriate gift at the completion of the experiment as token of appreciation. The study protocol was approved by the Institutional Review Board of Henan University, and all participants provided written informed consent prior to data collection.

**Table 1:** Descriptive characteristics of deaf children and hearing controls.

	**Deaf children**	**Hearing controls**
No. of children	31	28
Mean age (SD) (years)	11.613 (0.230)	11.321 (0.242)
Range of age (years)	9–13	9–13
Ratio of female/male (%)	48.39/51.61	53.57/46.43
With hearing aids/Without hearing aids (%)	48.39/51.61	//
Communication mode	Sign language	Oral language

### Stimuli and Procedure

**Figure 1:**
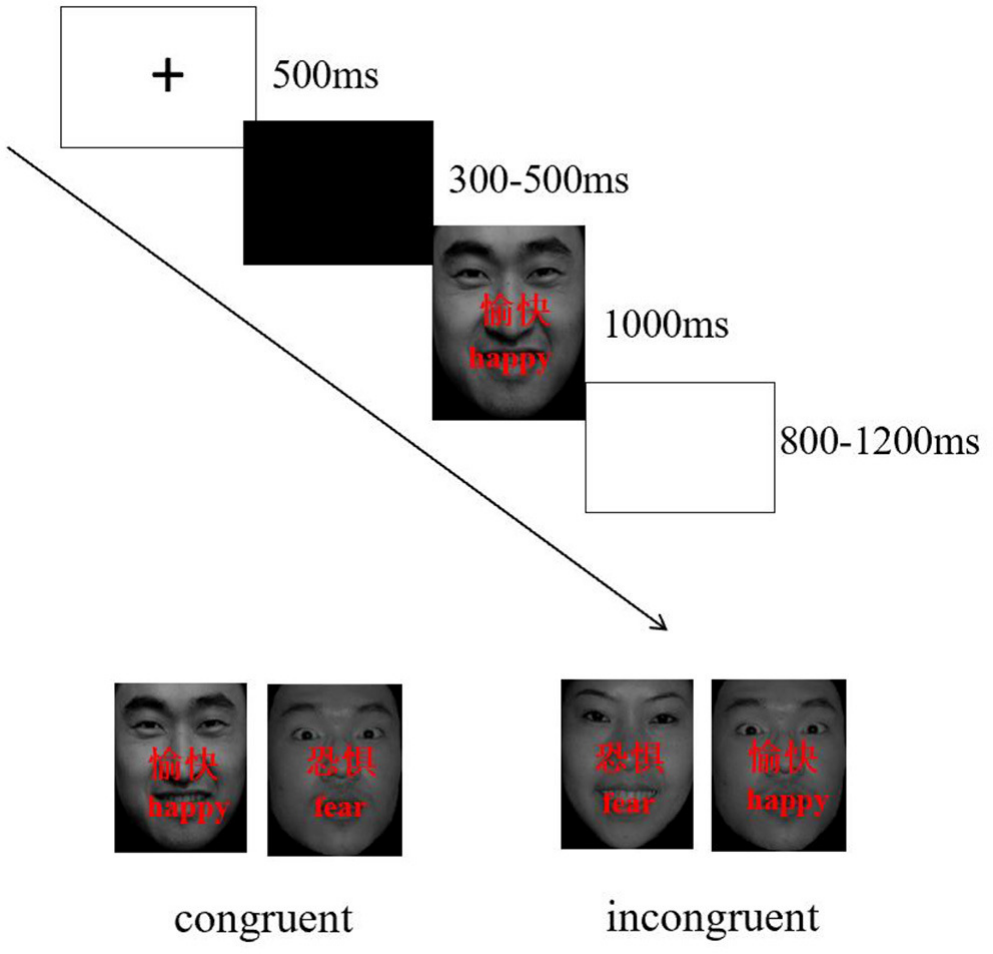
Procedures for emotional stroop task.

Twenty face pictures were selected from Chinese affective picture system (53), including 10 happy face pictures (5 female, 5 male) and 10 fearful face pictures (5 female, 5 male). Two Chinese characters, “愉快” (which means “happy”) or “恐惧” (which means “fear”) were superimposed on the faces in red. The words and facial expressions were either congruent (e.g., character meaning fear superimposed onto a fear face picture, see Figure 1) or incongruent (e.g., character meaning happy superimposed onto a fear face picture, see Figure 1). The stimuli were programmed by E-Prime 2.0 software, and they were presented on a Dell 19 -in monitor.

Participants performed modified face-word stroop task (judging facial expression) while sitting in quiet room with dim light, participants had to identify the facial expression of the target faces while ignoring the meaning of the words. They were instructed to respond by pressing a button, corresponding to “fear” faces (right index finger) or “happy” faces (right middle finger), as quickly and accurately as possible. The order of performing the experimental task was counterbalanced across participants.

The face-word stroop task consisted of 240 trials that were presented over 4 blocks (60 trials per block). Each block in each task consisted of an equal amount of congruent and incongruent trials. Stimuli were presented in random order within each block. Participants performed in a 24-trial practice block prior to the experiment. The timing and order of each trial was the same for each block: a fixation dot was presented for a specific duration (500 ms) followed by a blank screen of variable duration (300–500 ms). Then, the target face appeared for 1000 ms at the center of the screen. Participants had to respond within 1500 ms. The inter-trial interval (ITI) varied randomly between 800 ms and 1200 ms, with a mean of 1000 ms (Figure 1).

### EEG Recording

**Figure 2:**
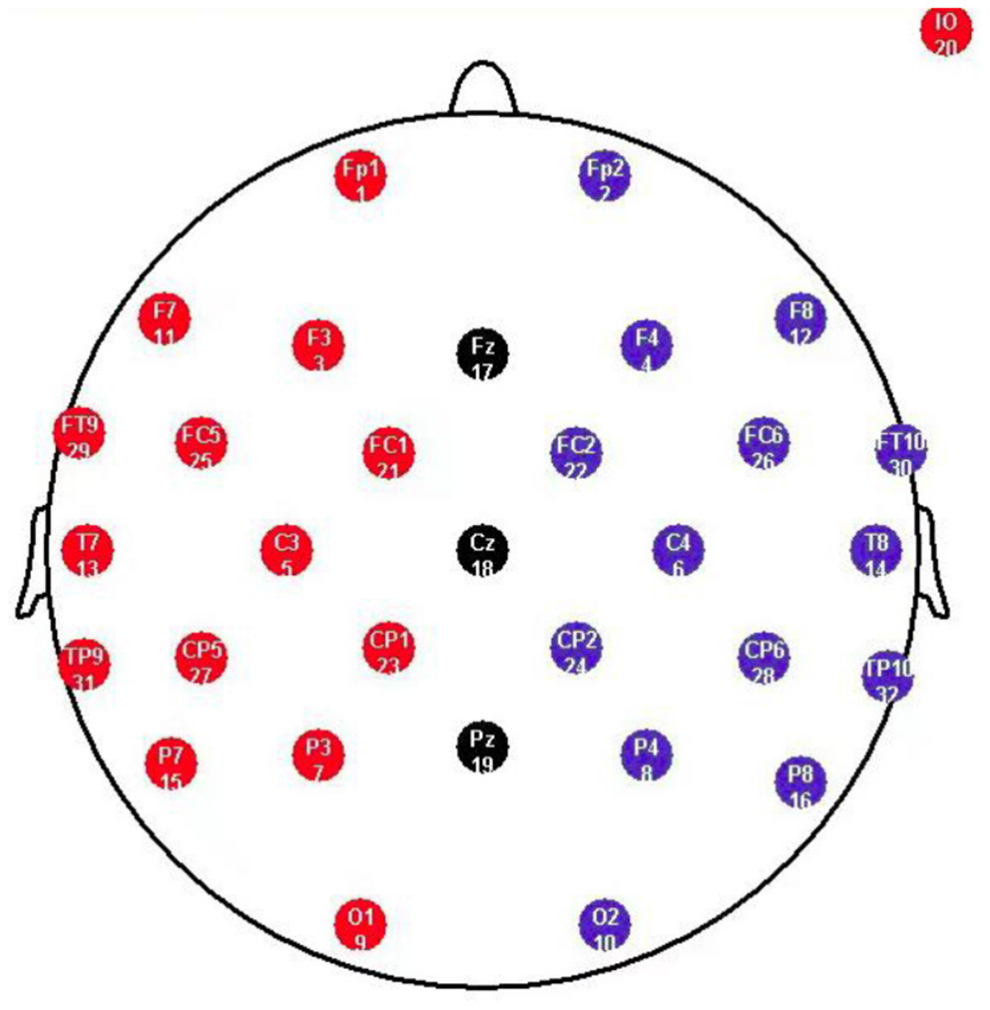
Standard electrode map, illustrating the commonly deployed 10-20 System. F refers to Frontal lobe, T refers to Temporal lobe, C refers to Central lobe, P refers to Parietal lobe, O refers to Occipital lobe, z refers to an electrode placed on the mid-line.

The electroencephalogram (EEG) was recorded from a 32 scalp standard channel cap (10/20 system; Brain Products, Munich, Germany) (Figure 2). Electrooculogram (EOG) was recorded from electrodes placed above the right eye. All electrode recordings were online referenced to FCz. All inter-electrode impedance was maintained below 5 kΩ. The EEG and EOG signals were amplified using a 0.01–100 Hz band pass filter and continuously sampled at 500 Hz/channel for offline analysis.

After data acquisition, EEG data were transferred into the EEGLAB and Letswave toolboxes, which are open-source Matlab toolboxes for neurophysiologic data analysis (54, 55). EEG were re-referenced to the average of the two mastoids and filtered with a band pass of 0.1–30 Hz. Epochs were extracted between the 200 ms pre-stimulus and 1000 ms post-stimulus time points, and the baseline correction was performed in 200 ms pre-stimulus interval. Eye movement artifacts were removed with ICA. Finally, data were inspected and cleansed manually for any obvious remaining artifacts.

### ERP Analysis

This study analyzed the potentials of the ERP components N1 and N450. The electrodes for further analysis were chosen according to ERP topographical distribution and previous studies (56, 57). Specifically, the amplitudes of the N1 (100-200 ms) were analyzed at F3, F4, Fz, and the N450 (330–400 ms) at C3, C4, Cz, P3, P4, Pz, and were measured as mean values. The time windows were determined through visual detection in the grand-averaged ERPs.

### Time–Frequency Analyses

An estimate of the oscillatory power as a function of time and frequency (time–frequency representation) was obtained from single-trial EEG epochs using the continuous wavelet transform (CWT) (55). The time–frequency representations were explored between 1 Hz and 30 Hz in steps of 0.29 Hz. Epochs were extracted between the 400 ms pre-stimulus and 1000 ms post-stimulus time points. To avoid edge effects when performing CWT, the pre-stimulus time interval (−400 ms to −200 ms) was used as a baseline interval. Based on average condition contrast maps and previous studies (58), 2 clusters were tested in this study: 4–7 Hz at 200–400 ms for theta (F3, F4, Fz, C3, C4, and Cz), 8–14 Hz at 600–800 ms for alpha (P3, P4 and Pz). Each of these oscillatory components was quantified as the mean amplitude within these time windows of each participant.

### Statistical Analysis

SPSS 20.0 was used to perform ANOVA or the chi-square test to investigate whether the demographic factors (including age and gender) showed significant differences between groups (deaf children and hearing controls). Furthermore, repeated measures ANOVA was conducted on behavioral and ERP data with group (deaf children vs. hearing controls) as a between-subject factor, while stimulus type (congruent, and incongruent), Hemisphere (Hemi) (only in EEG data: Left, Midline and Right) and antero-posterior distribution (AP) (only in EEG data: Frontal, Central, Parietal and Occipital) were considered as within-subject factors. For all the analyses in this study, the *p*-values were corrected by Greenhouse-Geisser correction when appropriate.

## Results

### Behavioral Data

#### Accuracy

ANOVA showed significant main effect of group on response accuracy (F_1, 57_ = 11.705, *p* = 0.002, η^2^ = 0.163), with overall lower accuracy in deaf children compared to hearing controls (see Table 2), which indicated that deaf children had difficulties in suppressing irrelevant information and suffered from deficient cognitive control mechanisms (4, 5, 50). The main effect of condition was also significant (F_1, 57_ = 63.094, *p* = 0.000, η^2^ = 0.525), with incongruent condition (0.872 ± 0.010) being significantly lower than congruent condition (0.927 ± 0.009), which indicated that in the presence of a conflict effect, the incongruent condition invested more cognitive resources than the congruent condition (28, 29, 59).

**Table 2:** Mean accuracy and reaction time (M ± SD) of deaf children and hearing controls, and results of repeated measures ANOVA for conditions.

	**Congruent**	**Incongruent**	**F_**GROUP**_ (*p*)**	**F_**CON**_ (*p*)**	F_CON*GROUP_ (*p*)
**ACC**
Total (*n* = 59)	0.927 ± 0.009	0.872 ± 0.010	63.094 (0.000^***^)	11.075 (0.002^***^)	1.696 (0.198)
Deaf Children (*n* = 31)	0.893 ± 0.012	0.847 ± 0.014			
Controls (*n* = 28)	0.961 ± 0.013	0.897 ± 0.014			
**Reaction Time (ms)**
Total (*n* = 59)	759.818 ± 14.833	816.231 ± 14.481	3.395 (0.071)	135.774 (0.000^***^)	24.981 (0.000^***^)
Deaf Children (*n* = 31)	745.278 ± 20.437	777.492 ± 19.952	23.325 (0.000^***^)		
Controls (*n* = 28)	774.358 ± 21.504	854.969 ± 20.994	131.910 (0.000^***^)		

*** *p < 0.01*.

#### Reaction Time

The significant main effect of condition was found that congruent condition (759.818 ± 14.833) was significantly faster than incongruent condition (816.231 ± 14.481) (F_1, 57_ = 135.774, *p* = 0.000, η^2^ = 0.704), which is consistent with the response accuracy, indicating that the conflict condition requires more cognitive resources and that participants need longer reaction times to make judgments. The interaction between group and condition was significant (F_1, 57_ = 24.981, *p* = 0.000, η^2^ = 0.305). Further analysis indicated that a significant interference effect with the congruent condition (745.278 ± 20.437) (774.358 ± 21.504) being faster than the incongruent condition (777.492 ± 19.952) (854.969 ± 20.994) in deaf children (F_1, 57_ = 23.325, *p* = 0.000, η^2^ = 0.290) and hearing controls (F_1, 57_ = 131.910, *p* = 0.000, η^2^ = 0.698).

#### ERP Amplitude Analysis

**Figure 3:**
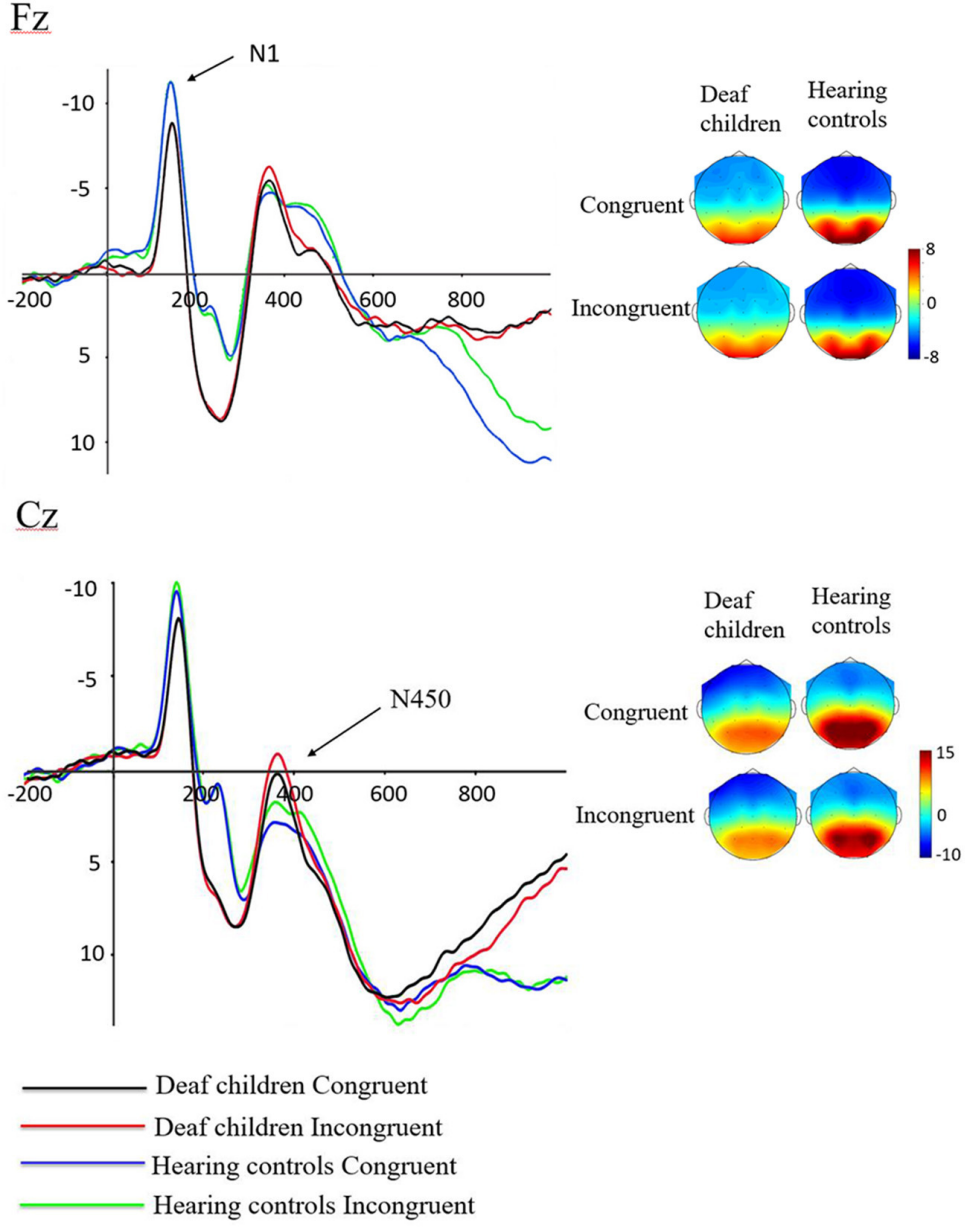
Waveforms of N1 and N450 components in emotional stroop task of deaf children and hearing controls.

For the consideration of space, we only included significant results in this part. Figure 3 showed the grand-averaged waveforms of deaf children and hearing controls. The Means and SEs for each component were displayed in Table 3, and the Means and SDs of amplitudes of each electrode N1 and N450 were displayed in Table 4.

**Table 3:** ERP amplitudes (M ± SD) of deaf children and hearing controls, and results of repeated measures ANOVA for conditions.

	**Congruent**	**Incongruent**	**F_**CON**_ (*p*)**	**F_**GROUP**_(*p*)**	F_CON*GROUP_ (*p*)
**N1 amplitude**
Total (*n* = 59)	−6.898 ± 0.574	−6.776 ± 0.496	0.118 (0.732)	4.517 (0.038^*^)	0.171 (0.681)
Deaf Children (*n* = 31)	−5.896 ± 0.791	−5.627 ± 0.683			
Controls (*n* = 28)	−7.900 ± 0.832	−7.925 ± 0.719			
**N450 amplitude**
Total (*n* = 59)	7.285 ± 0.938	6.319 ± 0.945	5.090 (0.028^*^)	5.883 (0.018^*^)	0.237 (0.629)
Deaf Children (*n* = 31)	4.957 ± 1.293	4.199 ± 1.302			
Controls (n=28)	9.613 ±1.360	8.439 ±1.370			

* *p < 0.05*.

**Table 4:** The average amplitudes (μV) of the ERP components (M ± SD) between deaf children and hearing controls.

**ERP components**	**Stimulus type**	**Electrode point**	**Average amplitudes (μV)**	
			**Deaf children**	**Hearing controls**
N1	congruent	F3	−6.066 ± 4.142	−7.687 ± 4.475
		Fz	−5.703 ± 4.479	−8.360 ± 4.716
		F4	−5.919 ± 4.569	−7.654 ± 4.570
	incongruent	F3	−5.710 ± 3.381	−7.682 ± 3.868
		Fz	−5.681 ± 3.492	−8.360 ± 4.257
		F4	−5.489 ± 4.195	−7.733 ± 4.257
N450	congruent	C3	−0.395 ± 7.057	3.522 ± 7.800
		Cz	1.233 ± 7.891	3.107 ± 10.568
		C4	1.171 ± 8.083	3.162 ± 8.849
		P3	8.555 ± 7.166	15.931 ± 8.604
		Pz	9.506 ± 6.065	15.075 ± 9.569
		P4	9.670 ± 7.649	16.884 ± 9.240
	incongruent	C3	−0.818 ± 7.630	2.660 ± 7.194
		Cz	0.182 ± 8.320	2.191 ± 10.376
		C4	0.466 ± 8.285	2.414 ± 8.185
		P3	8.075 ± 7.098	14.529 ± 7.968
		Pz	8.449 ± 7.165	13.514 ± 9.028
		P4	8.840 ± 7.791	15.327 ± 8.594

#### N1

A repeated-measures analysis of variance (ANOVA) was applied in this procedure with N1 amplitude as dependent variable, with stimulus type (congruent, and incongruent), Hemisphere (Hemi: Left, Midline and Right) and AP [frontal(F) (electrodes: F3, Fz, F4), central (C) (electrodes: C3, Cz, C4), and parietal (P) (electrodes: P3, Pz, P4)] as within-subject factors, and group (deaf children vs. hearing controls) as a between-subject factor. Results showed significant main effect of group on N1 amplitude (F_1, 57_ = 4.517, *p* = 0.038, η^2^ = 0.073), with hearing controls (−7.913 ± 0.734 μV) eliciting overall larger N1 compared to deaf children (−5.761 ± 0.697 μV), which suggested that the smaller N1 amplitudes were neurophysiological reflex of deficient inhibition for emotional stroop task in deaf children (60). The interaction effect of Group × Hemisphere was also significant for N1 amplitude (F_2, 56_ = 3.197, *p* = 0.046, η^2^ = 0.053). Further analysis indicated that midline region (Fz) elicited larger N1 activation in hearing controls (−8.360 ± 0.75 6μV) compared to deaf children (−5.692 ± 0.718 μV), while there was no significant group difference in response to right (F4) and left hemispheres (F3).

#### N450

Analysis of N450 amplitude showed a significant main effect of group (F_1, 57_ = 5.883, *p* = 0.018, η^2^ = 0.094), with deaf children (4.578 ± 1.263 μV) eliciting overall larger N450 compared to hearing controls (9.026 ± 1.329 μV). There also was a significant main effect of condition (F_1, 57_ = 5.090, *p* = 0.028, η^2^ = 0.082), incongruent condition (6.319 ± 0.945 μV) eliciting larger N450 than congruent condition (7.285 ± 0.938μV). The results of the N450 amplitudes were consistent with behavioral outcomes, reflecting deficits in inhibitory control in deaf children and inconsistent conditions requiring more effort to complete. The results showed a significant main effect of AP (F_1, 57_ = 203.155, *p* = 0.000, η^2^ = 0.781), with central area (1.574 ± 1.031 μV) eliciting larger N450 than parietal area (12.030 ± 0.942 μV). There was also a significant Group × AP interaction effect (F_1, 57_ = 6.798, *p* = 0.012, η^2^ = 0.107). According to further analysis, this interaction indicated that the central area (0.306 ± 1.420 μV) (2.842 ± 1.495 μV) elicited larger N450 than parietal area (8.849 ± 1.298 μV) (15.210 ± 1.366 μV) in both deaf children (F_1, 57_ = 71.447, *p* = 0.000, η^2^ = 0.556) and hearing controls (F_1, 57_ = 135.262, *p* = 0.000, η^2^ = 0.704).

### Time-Frequency Results

#### Theta Activity

**Figure 4:**
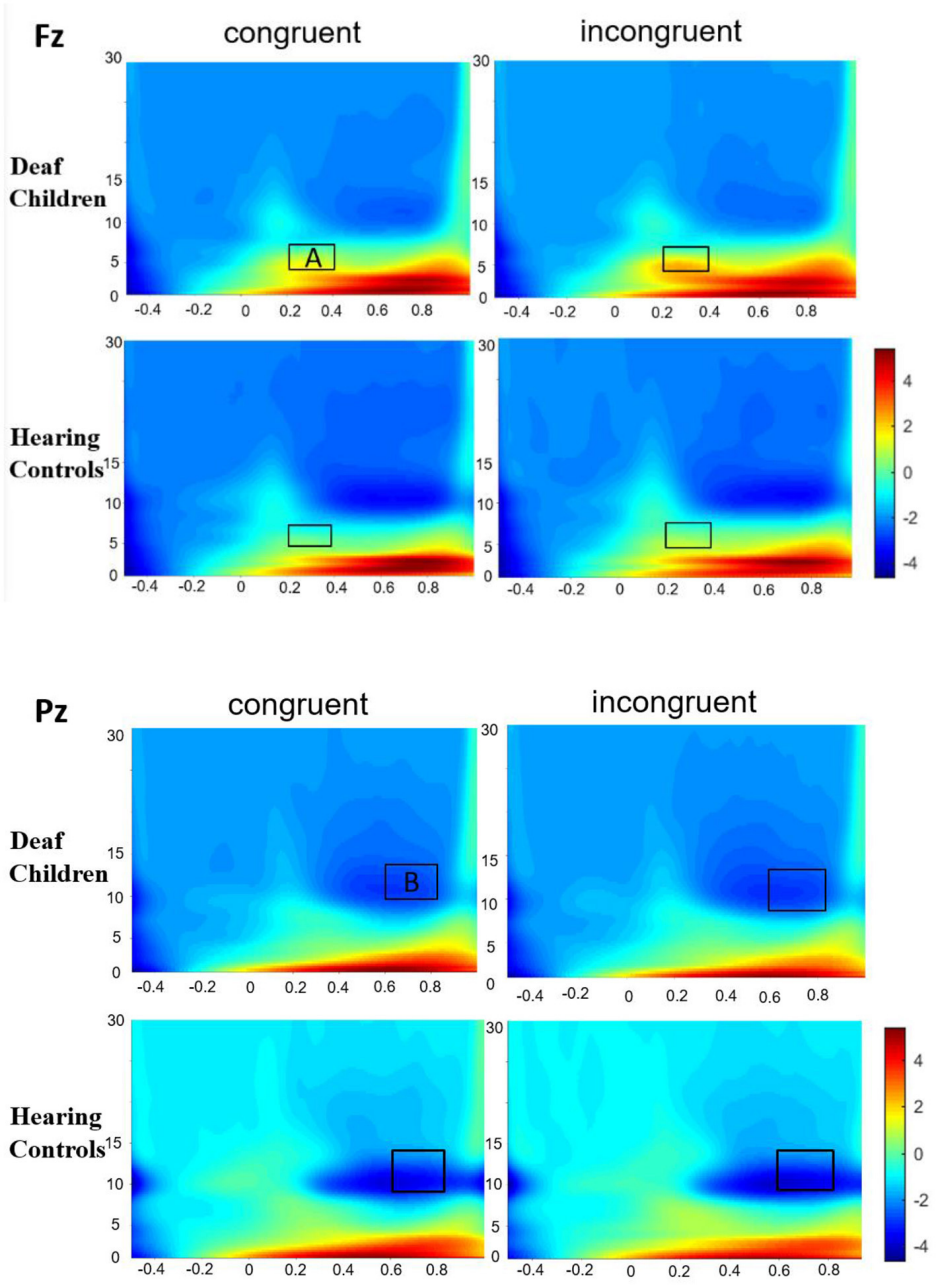
Group-averaged time-frequency spectrogram during facial emotion recognition. Time (in ms) is denoted on the x-axis, with 0 ms defined as the onset of the stimuli. Frequency (in Hz) is shown on the y-axis. A represent the theta band (200–400 ms), and B represent the alpha band (600–800 ms).

Figure 4 presented the Time-frequency (TF) analysis results. Theta synchronization (200–400 ms) showed significant main effect of condition (F_1, 57_ = 5.022, *p* = 0.029, η^2^ = 0.081), with incongruent condition (7.135 ± 0.467) eliciting larger theta synchronization than congruent condition (6.624 ± 0.460). Theta synchronization also showed significant main effect of group (F_1, 57_ = 5.348, *p* = 0.024, η^2^ = 0.086), with deaf children (7.920 ± 0.620) eliciting larger theta synchronization than hearing controls (5.840 ± 0.652), which were consistent with the results of N450. There also was a significant main effect of emotional background (F_1, 57_ = 7.659, *p* = 0.008, η^2^ = 0.118), with fear background (7.189 ± 0.458) eliciting larger theta synchronization than happy background (6.571 ± 0.469). The results also showed significant main effect of AP (F_1, 57_ = 44.420, *p* = 0.000, η^2^ = 0.438) and hemisphere (F_2, 56_ = 12.970, *p* = 0.000, η^2^ = 0.185), with frontal area (7.674 ± 0.490) eliciting larger theta synchronization than central area (6.085 ± 0.439), midline region (Fz, Cz) (7.408 ± 0.494) eliciting larger theta synchronization than left hemispheres (F3, C3) (6.857 ± 0.476) and right hemispheres (F4, C4) (5.840 ± 0.652). A significant interaction was found between the emotional background and group (F_1, 57_ = 7.832, *p* = 0.007, η^2^ = 0.121), further analysis indicated that deaf children (8.541 ± 0.631) elicited larger theta synchronization in fear background compared to hearing controls (5.836 ± 0.663), while there was no significant group difference in response to happy background.

#### Alpha Activity

Alpha desynchronization (600–800 ms) showed significant main effect of group (F_1, 57_ = 6.868, *p* = 0.011, η^2^ = 0.108), with hearing controls (−6.376 ± 1.003) eliciting larger alpha desynchronization than deaf children (−2.750 ± 0.953), which were in line with the results of N1. Alpha desynchronization also showed significant main effect of emotional background (F_1, 57_ = 6.939, *p* = 0.011, η^2^ = 0.109), with fear background (−4.882 ± 0.773) eliciting larger alpha desynchronization than happy background (−4.244 ± 0.624). There also was a significant main effect of hemisphere (F_2, 56_ = 10.817, *p* = 0.000, η^2^ = 0.160), with midline region (Pz) (−3.665 ± 0.540) eliciting smaller alpha desynchronization than left hemispheres (P3) (−4.823 ± 0.798) and right hemispheres (P4) (−5.190 ± 0.790).

## Discussion

The present study explored the emotional interference effect among deaf children. Behavioral results showed that the main effect of condition was significant not only for the accuracy data but also for the reaction time data, which indicated that in the presence of a conflict effect, the incongruent condition invested more cognitive resources than the congruent condition (28, 29, 59). In addition, it was also found that deaf children demonstrated significantly lower accuracy rate in emotional stroop task than hearing controls, which is consistent with previous findings that deaf children had difficulties in suppressing irrelevant information and suffered from deficient cognitive control mechanisms (4, 5, 50).

These behavioral results were further explored by ERP analysis. Deaf children showed diminished activation in the emotional interference processing components compared to hearing controls. Given that the N1 components reflect the attentional focus on the target and a discrimination process within the focus of attention (31, 60), the smaller N1 amplitudes suggest two important points. First, although the emotional stroop task needed participants to concentrate on the facial expression of the picture, the deaf children paid less attention on the task-relevant information (the words of the picture) because of the interference of the meaning of the words. Therefore, a weaker allocation for target information was obtained. Second, for task-irrelevant information (the words of the picture), the deaf children also automatically put attentional resources on it; consequently, the attentional resources for task completing were deficient, and the time needed to complete the task was prolonged or the task was poorly performed. So the smaller N1 amplitudes might be correlated with a slower response to emotional interference stimuli, which suggested that the smaller N1 amplitudes were neurophysiological reflex of deficient inhibition for emotional stroop task (61). This finding is in accordance with the evidence of similar alterations during stroop task in individuals with schizophrenia, amblyopic, obsessive compulsive disorder, or depression (31, 38, 62, 63).

In contrast to N1, deaf children showed enhanced activation in N450 compared to hearing controls. According to previous studies, the N450 is a valid index of conflict monitoring in emotional conflict control tasks and shows larger negative amplitude in the incongruent condition compared to congruent condition (34–37, 64). The role of the anterior cingulate cortex (ACC) and the dorsolateral pre-frontal cortex (DLPFC) regions have been shown to be components of a neural network which plays a critical role in the completion of tasks requiring self-monitoring and inhibition (21). The N450 component demonstrated enhanced amplitudes in deaf children, suggesting that deaf children may require a greater recruitment of cognitive resources from the ACC and DLPFC to achieve the performance levels of hearing controls during emotional stroop task (22, 64). In addition, compared to healthy controls, major depressive disorder (MDD) patients showed enhanced N450 amplitude (31, 65). Patients with attention deficit hyperactivity disorder (AHDH), nocturnal enuresis (NE) and developmental coordination disorder (DCD) showed increased activation in the bilateral temporoparietal junctions, bilateral dorsolateral pre-frontal cortex, and bilateral anterior cingulate cortex (66–68). There is a causal relationship between the decreased N1 and the increased N450, which is similar to previous studies of person with depression (31). The correlation may be due to reduced early attention requiring more effort later in emotional stroop task. Combined with the analysis of behavioral results which showed that accuracy rate of deaf children in emotional stroop task was significantly lower than hearing controls, it can be found that although deaf children made more efforts and showed more activation on N450 than hearing controls, they did not reach the same level as hearing controls, which revealed the emotional impairment of cognitive monitoring function of the deaf children, meanwhile indicated that the emotional cognitive resources for monitoring conflict information and inhibition irrelevant information in the inhibition control process of the deaf children are very limited.

Besides ERP analysis, the current study employed time–frequency measures in alpha and theta band which showed significantly more desynchronization in hearing controls and significantly more synchronization in deaf children and incongruent condition. A number of studies have found that alpha oscillation was a reliable marker of attention (69, 70), theta band was related with central executive and working memory processes, and reflected initiation of the central executive processes to detect interference and to inhibit the response for task-irrelevant features (40, 42, 43). Alpha desynchronization was similar to the results from ERP components in N1. Previous studies have showed diminished alpha suppression in the predominantly inattentive (IA) and absent alpha oscillation in ADHD (71, 72). Therefore, the diminished alpha desynchronization might suggest impaired attention distribution ability during emotional interference processing of deaf children. Theta synchronization also showed a conflict effect which was in line with the previous studies (73, 74). In addition, consistent with ERP results, deaf children showed enhanced theta synchronization which might suggest impaired cognitive monitoring function during emotional interference processing of them.

The negative impact poor inhibitory control has on a range of outcomes for deaf children, but considering that after a period of training, children showed great improvements in their inhibition skills (49, 75). Therefore, we can train the inhibitory control of emotional interference ability of deaf children to help them form a healthy personality and better integrate into the society. Specifically, school education can strengthen the training of deaf children's inhibitory control of emotional interference ability through flexible and diverse classroom forms. Based on the fact that deaf children are more inclined to visual images when receiving information, they have stronger perception and memory for actions, expressions or visualized pictures. Teachers can use multimedia animation and small games in teaching to mobilize students to participate in various emotional situations, help them improve the problems in emotional control, let them understand the way of emotional expression, enhance their ability to understand the behavior intention of others, and have a certain ability to predict the behavior consequences, so as to improve their ability of the inhibitory control of emotional interference.

## Contributions, Limitations, and Future Directions

Combining the ERP and TFA data analysis methods simultaneously can not only enhance the energy of ERP components by using the high temporal resolution ERP technology, but also greatly reduce the amplitude and noise of the spontaneous EEG, so as to reveal the time process of individuals in the process of inhibitory control of emotional interference (76). Moreover, time-frequency analysis can be used to simultaneously extract the temporal and spectral domains of event-related brain activity, improving the detectability of ERP and allowing characterization of non-phase-locked components that cannot be identified by traditional time-domain averaging in healthy and deaf children (77, 78).

The limitation of this study is that it does not use nuclear magnetic technology with high spatial resolution, but only ERP technology with high temporal resolution. Future research should use the combination of ERP and functional magnetic resonance imaging (fMRI) to measure the neural mechanism of deaf children in emotional stroop task, and use the advantages of the combination of high temporal resolution and high spatial resolution to explore the damage degree of inhibitory control of emotional interference and the corresponding impaired brain regions of deaf children.

## Conclusion

In conclusion, the current study revealed major deficits in deaf children during emotion-related conflict control, with overall worse behavioral performance and reduced activation of N1 and alpha desynchronization, and enhanced activation of N450 and theta synchronization compared to the hearing controls, which might suggest impaired attention allocation ability and cognitive monitoring function during conflict detection process in deaf children. The Findings enriched the understanding of impaired inhibitory control of emotional interference in deaf children, and helped educators to take timely and appropriate intervention measures to promote the optimal neuropsychological development of deaf children.

## Data Availability Statement

The raw data supporting the conclusions of this article will be made available by the authors, without undue reservation.

## Ethics Statement

The studies involving human participants were reviewed and approved by Institutional Review Board of Henan University. Written informed consent to participate in this study was provided by the participants' legal guardian/next of kin.

## Author Contributions

JZ contributed to the conception of the study. HG contributed significantly to analysis and manuscript preparation. QC performed the data analyses and wrote the manuscript. XL helped revise the manuscript. QC contributed to the interpretation and discussion of the results of the analysis. All authors contributed to the article and approved the submitted version.

## Funding

This work was supported by the Science and Technology Research Project of Henan Provincial Department of Science and Technology [212102310985], the Humanities and Social Science Research Project of Henan Provincial Department of Education [2020-ZDJH-026], and the Social Science Planning Project of Henan Province [2021CJY051].

## Conflict of Interest

The authors declare that the research was conducted in the absence of any commercial or financial relationships that could be construed as a potential conflict of interest.

## Publisher's Note

All claims expressed in this article are solely those of the authors and do not necessarily represent those of their affiliated organizations, or those of the publisher, the editors and the reviewers. Any product that may be evaluated in this article, or claim that may be made by its manufacturer, is not guaranteed or endorsed by the publisher.
